# Rationing conscience

**DOI:** 10.1136/medethics-2016-103795

**Published:** 2016-10-12

**Authors:** Dominic Wilkinson

**Affiliations:** 1 Faculty of Philosophy, Oxford Uehiro Centre for Practical Ethics, University of Oxford, Oxford, UK; 2 Newborn Care Unit, John Radcliffe Hospital, Oxford, UK

**Keywords:** Conscientious Objection, Resource Allocation

## Abstract

Decisions about allocation of limited healthcare resources are frequently controversial. These decisions are usually based on careful analysis of medical, scientific and health economic evidence. Yet, decisions are also necessarily based on value judgements. There may be differing views among health professionals about how to allocate resources or how to evaluate existing evidence. In specific cases, professionals may have strong personal views (contrary to professional or societal norms) that treatment should or should not be provided. Could these disagreements rise to the level of a conscientious objection? If so, should conscientious objections to existing allocation decisions be accommodated? In the first part of this paper, I assess whether resource allocation could be a matter of conscience. I analyse conceptual and normative models of conscientious objection and argue that rationing could be a matter for conscience. I distinguish between negative and positive forms: conscientious non-treatment and conscientious treatment. In the second part of the paper, I identify distinctive challenges for conscientious objections to resource allocation. Such objections are almost always inappropriate.

## Introduction

While controlled allocation of scarce resources (‘rationing’) appears to be inevitable in health systems,^1^ ^2^, ^(pp 14–19)^ health professionals do not always agree about the right way to allocate those resources.^3–5^ Those disagreements can take different forms. Consider the following cases:

### Case 1^i^

Dr A is counselling parents following antenatal diagnosis of a major chromosomal disorder. The fetus also has evidence of congenital heart disease. Babies with this chromosomal disorder have a high chance of dying in the newborn period, and will be severely disabled if they survive. There is no official policy not to provide cardiac surgery for infants with this chromosomal disorder. Some infants with this disorder receive cardiac surgery and long-term survival following surgery has been described. However, in Dr A's view, cardiac surgery would be unethical in this situation; even if it were potentially beneficial, it would constitute an unjust use of limited medical resources. Dr A does not discuss the option of cardiac surgery with the parents.

### Case 2^ii^

An elderly patient in a persistent vegetative state and multi-organ failure has been in intensive care for a prolonged period. Medical staff believe that continued intensive care would be futile, and that treatment should be withdrawn. However, his family wishes treatment to continue and obtains a court order for treatment to continue pending review. Several doctors in the intensive care unit refuse to be involved in treatment of the patient because they regarded it as unethical.

### Case 3^iii^

A couple, Jane and Peter, is having trouble conceiving. Regional guidelines specify that publicly funded fertility treatment will only be available to women with a body mass index (BMI) in the normal range. Jane is 36 and has a BMI of 39. She has tried to lose weight over a prolonged period without success. Other fertility treatment centres have declined to provide in vitro fertilisation (IVF); however, her new fertility specialist believes that the restrictions on fertility treatment for obese women are unjust.^iv^ She provides fertility treatment to Jane and Peter.

### Case 4^v^

Mrs L presents seeking a mammogram. She is concerned about breast cancer as her younger sister was recently diagnosed with this condition. Physical examination is normal. From review of the medical literature, Mrs L's doctor believes that an annual mammogram would be indicated; however, Mrs L's insurer will not cover the test. Mrs L is of limited means, and will not be able to pay for the mammogram. Her doctor regards the insurance limitations as unfair. She documents ‘suspicious breast lump’ on Mrs L's insurance claim form (though this is not accurate).^vi^

In the cases above, doctors did not use the language of ‘conscientious objection’ (CO). Nevertheless, the nature of the concerns and the action taken by the professionals might be thought to at least share a family resemblance with standard cases of COs in medicine.^13^, ^(p 8)^ If professional disagreements about rationing *can* count as CO, this would be significant, since such objections are often granted special status and regarded as deserving of respect and accommodation by the law and professional bodies.^13^

For the sake of this paper, I will set aside substantive questions about how resources should be allocated. I will assume that some form of rationing is permissible. I will also set aside the wider questions about COs, and assume that COs in healthcare should, at least sometimes, be accommodated. I will focus particularly on resource allocation in public healthcare systems. As case 4 highlights, however, similar issues may apply to professional objections to allocation decisions by insurers or managed care organisations.^14^ Finally, while acknowledging that in real cases clinicians might have several different reasons for objecting to a particular treatment option, I will concentrate on those concerns that relate to resource allocation per se.^vii^

## Conscientious rationing

### Conceptual questions

There are a series of questions that might be asked. First, are objections to resource allocation consistent with the concept of COs?

There are different accounts of what it means to have a CO. Some are relatively restrictive. For example,

CO(narrow definition): an objection to provide a good or service based on a sincerely held set of moral convictions arising from belief in and relation to a Supreme Being or arising from a belief that has a parallel place in the individual's life to that filled by God amongst religious adherents.^13^, ^(p 3)^

This narrow definition of CO may exclude cases of objection to resource allocation since such objections would not usually relate to religious or quasi-religious beliefs.^viii^ However, objections to abortion or physician-assisted dying do not necessarily relate to beliefs of that kind either, so this definition may be too restrictive. An alternative account identifies CO as refusals to provide a good or service on the basis that this would be incompatible with the agent's core moral beliefs.^13^, ^(p 4–5)^ It is plausible that objections to particular allocation decisions could represent core beliefs. For example, in case 2, one professional's resignation letter described continued treatment as ‘an abomination’, and wrote ‘I can't do it’, appearing to indicate the depth of feeling associated with objections to treatment.^17^ However, some have argued that judgements about the futility of medical treatment represent professional judgements, and should not count as CO.^13^, ^(p 6)^

A recent professional guideline on COs set aside the source or quality of beliefs to focus instead on actions that would be contrary to either personal or professional beliefs:

CO (broad definition): Objections to providing legal, professionally accepted, and otherwise available medical services based on a clinician's judgment that to do what is requested would be morally wrong.^18^

All of the above cases involved physicians' moral disagreement with allocation decisions. Some of them, though (cases 3 and 4), led to positive actions rather than to withholding treatment. Should these also count as forms of CO? It is beyond the scope of this paper to address differences between negative and positive duties. However, Mark Wicclair has argued persuasively that there are no good reasons to selectively protect negative claims of conscience.^19^ If positive claims of conscience are included too, it may allow CO to be claimed in cases 3 and 4. (Wicclair leaves open the question of whether positive and negative conscience claims are completely symmetric and should be treated equally. In the context of resource allocation, there are reasons to treat these differently (see below).) For clarity, here are two different forms of CO to resource allocation:

Conscientious Resource-based Non-treatment (Conscientious Non-treatment): A considered decision not to provide a legally and professionally accepted medical treatment on the basis of a personal belief that this would be an unjust use of limited healthcare resources

Conscientious Resource-based Treatment (Conscientious Treatment): A considered decision to provide a legally available medical treatment on the basis of a personal belief that this would be justified, despite a professional norm that because of limited healthcare resources the treatment should not be provided

Conscientious non-treatment or treatment might take place in the absence of a clear guideline or policy about treatment (case 1); however, they are most likely to be identified where they are in contravention of an existing guideline or policy (eg, cases 3 and 4).

### Normative questions

There are several reasons that are commonly given for accommodating COs. Do these apply to cases of disagreement about rationing?

Four commonly cited reasons in favour of accommodating COs include protecting clinicians’ moral integrity, respecting their autonomy, improving the quality of medical care (particularly through allowing diversity) and identifying needed changes in professional norms.^18^ All of these could straightforwardly be applied to instances of professional disagreement about resource allocation. For example, the clinicians who resigned in case 2 clearly felt that their personal moral integrity was threatened by continuing to provide treatment. In cases 1 and 4, permitting the doctors to make a determination about admission to intensive care, or the appropriateness of screening mammography would respect their personal and professional freedom to make decisions about medical treatment. It might be argued that decisions about the appropriateness of different treatment options are a fundamental example of professional autonomy.

Furthermore, allowing doctors to object to resource allocation decisions would potentially encourage them to take engage with resource allocation questions and determine how best to manage limited resources. It would arguably promote sensitivity among professionals to the claims of their patients, and avoid a sense that they are merely enforcing rules laid down by others. Finally, accommodating COs to resource allocation might help to identify where existing allocation schemes should be modified. For example, if a large number of professionals feel that screening mammography is appropriate and manipulate claim forms to ensure access, perhaps the insurer would be compelled to change its policy. If doctors elect to provide fertility treatment for a group of patients previously excluded, it may generate important data about the benefit (or non-benefit) of such treatment.

To sum up the arguments thus far, professional objections to extant resource allocation could be consistent with existing broad concepts of CO in healthcare—either in situations where professionals conscientiously ration treatment or provide it contrary to rationing policies. The ethical arguments that support accommodation of COs also potentially apply to cases of objection to allocation decisions. This suggests that doctors could conscientiously object to allocation decisions.

I have not argued that resource-based COs are identical to more traditional forms of CO in medicine nor that they are necessarily equally weighty. For example, Christopher Cowley claims that resource-based COs differ from other forms of CO (eg, abortion) in the type and directness of the harm caused if objections are not accommodated.^20^ This raises the possibility that CO to resource allocation would be treated differently from other forms of CO.

Are there particular features of resource allocation that would count against CO?

## Rationing conscience

Standard instances of CO in medicine represent a conflict between the wishes of the professional and the wishes of the patient. Cases of conscientious non-treatment might also have this character. However, conscientious treatment gives rise to a different conflict. In cases like 3 and 4 above, the professional's wishes coincide with those of the patient. Instead, such cases appear to represent a conflict between the wishes of the professional and those of wider society (and potentially of other patients).

Since the ethical considerations are different, we need to consider the two forms separately.

### Against conscientious non-treatment

Reasons that are provided against other forms of CO include that such objections violate core professional commitments, fail to protect vulnerable patients, create hardships for other clinicians (where accommodated) or are discriminatory.^18^ Some of these might be cited against conscientious non-treatment. For example, we might believe that the doctor's decision in case 1 conflicts with his duty to safeguard vulnerable patients, represents a form of invidious discrimination, or violates professional commitments.^18^ Yet, it would be arguably acceptable (and compatible with professional commitments) in case 1 or case 2 for the clinicians to withhold treatment if there were clear policies or guidelines supporting such an action. (Clearly, some who are opposed to rationing will dispute this. However, as noted earlier, I am focusing arguments in this paper on a setting in which resource allocation is an accepted (if regretted) feature of medical practice.)

What does seem concerning about the decisions in cases 1 and 2 is the apparent variation between clinicians in their responses to the case. It seems worryingly arbitrary that whether or not a patient is offered cardiac surgery, or intensive care is continued depends on which doctor happens to be on call. There appears to be a ‘roster lottery’ affecting the provision of potentially life-saving medical treatment.^21^

Concern about variation in decision making and in access to treatment might apply to other cases of CO. For example, it is likely to be of concern that some pharmacies will provide emergency contraception, while others will not. However, lack of consistency poses a particular problem for resource allocation^22^ since it seems to be contrary to all major theories of justly distributing scarce resources. Whether resources are allotted on the basis of greatest benefit, greatest clinical need or equal access, patients should only be treated differently if there are ethically relevant differences between them. The identity of the clinician does not appear to be ethically relevant.

Could a fair process of allocation yield variable decisions? Norm Daniels in ‘Just Health’ considers the hypothetical example of Jack and Jill, who have identical conditions and require an expensive cancer treatment.^23^, ^(pp 135–7)^ They apply to their respective health authority or health insurer for access to the treatment. One health authority (or health plan) approves the treatment for Jack, while the other declines Jill's request. Daniels argues that it is not necessarily unjust for the outcome of allocation to be different, as long as the process is consistent; fair decision-making *processes* may reach different conclusions in the setting of moral uncertainty. Moreover, there may be reasons to allow regional authorities (or insurers) to weigh up their priorities and allocate to different treatments as they see fit. However, such defences of regional variation in allocation decisions arguably do not apply to individual decision makers. Idiosyncratic determinations about available resources do not represent a fair decision-making process. Kristin Baeroe contends that some variation in microallocation decisions is inevitable, but proposes that physicians should strive towards common ground and a common basis for distinguishing between cases.^22^, ^(p 98)^ This aim for consistent decision making appears to preclude conscientious non-treatment.

### Against conscientious treatment

The positive form of CO to resource allocation might raise different concerns. The patients of IVF doctors who provide treatment outside conventional limits are unlikely to complain of inconsistency or unfairness. However, other women (whose doctors adhere to the guidelines) could complain. The often cited ‘publicity’ condition of fair allocation^24^ may require those other women to be informed, for example, that some doctors will provide IVF above the standard BMI limit and may lead them to seek that treatment. That would potentially defeat the purpose of making community-level decisions about allocation and imposing limits on treatment.^ix^

Partly as a consequence of this, conscientious treatment appears to be unfair in a different way, since it imposes the costs of CO on the wider community and would have implications on access to treatment for others. For example, the UK National Health Service does not routinely fund the breast cancer drug trastuzumab emtansine (Kadcyla), which costs approximately £90 000 to extend life by 6 months.^26^ This is because the cost significantly exceeds the usual cost-effectiveness threshold used in the UK to set a limit on affordable treatments (approximately £20–£30 000 per quality-adjusted life year saved). An individual doctor might disagree with the official assessment of Kadcyla, and choose to provide it for her patients with advanced breast cancer.^x^ Yet, this would then potentially limit the ability of the health service to provide other less expensive (and potentially more effective) treatments.

Could conscientious treatment be accommodated? One option would be to allow individual clinicians to provide the rationed treatment as long as it imposed no greater cost on the public health system than currently funded alternatives. This may require patients to partly or completely fund their treatment. However, while the latter would accommodate objections, it would potentially undermine the point of the objection. (The objections in cases 3 and 4 are to the public health system or the insurer failing to provide treatment. A solution that means that the patient is paying for treatment herself does not seem to actually accommodate the objections at all.) It may also require physicians to provide their services pro bono. Alternatively, it may be possible to give clinicians discretion to provide non-standard treatment, as long as the incremental cost is within a reasonable limit. This may permit conscientious treatment only in a small subset of cases.

## Conclusions

COs manifest when individuals face a conflict between their own values and what they are being asked or required to do. In healthcare, it is often felt to be important to respect the different ethical viewpoints of professionals and therefore to accommodate COs to morally controversial treatment options. Since resource allocation decisions are frequently contentious, and involve value judgements, it might be anticipated that these could give rise to COs. In this paper, I have argued that COs to allocation decisions are consistent with broad concepts of CO, and that the arguments in favour of accommodating CO would also apply to conscientious non-treatment or conscientious treatment. However, I have also outlined substantial arguments against accommodating CO to allocation; such accommodation would almost always be inappropriate. Conscientious non-treatment or treatment run counter to fundamental principles of allocation including consistency, and the need to impose limits on available treatment.

## Footnotes

i This is a composite version of real cases encountered. For examples of the disagreement caused by cases like this, see Gali^6^ and Janvier.^7^ As another example of this type of objection, Savulescu imagines a doctor who is convinced by the ‘fair innings’ argument and decides not to admit patients over the age of 70 to the intensive care unit.^8^

ii This is an abbreviated version of a real published case.^9^

iii This case is based on a real one.^10^ Note that in the real case treatment was provided by a private clinic rather than within the public health system.

iv In this case, the fertility specialist might object to non-provision of treatment either because it is discriminatory (against obese patients) or because it is based on mistaken assessment of the cost/benefit, or both. For the purposes of this paper, either might form the basis of a conscientious objection.

v Thirty-four per cent of US physicians were prepared to lie on an insurance claim form in a survey version of this case.^11^

vi Tavaglione and Hurst defend ‘conscientious’ deceit on behalf of the patient in non-ideal settings like this case.^12^ Note that individuals who are dishonest might not be regarded as conscientious objectors. For example, people who lie about their health to avoid being drafted for war are viewed differently from those who publicly refuse to serve based on core personal or religious values. However, it depends on whether it is possible to avoid military service by telling the truth. In countries where there is no recognised mechanism for registering a CO and opting out of military service, someone might dissemble because that is the only way of acting in accordance with their conscience. Analogously, Mrs L's doctor knows that if he tells the truth on the claim form that she will not have the investigation. His decision to lie is motivated by his conscience-based objection to the insurer's allocation decision. I am grateful to a reviewer for this point.

vii In cases of ostensibly futile treatment, such as case 1 or 2, clinicians might have concerns about harm to patients, or about limited resources.^15^ I will concentrate on the latter. We could imagine in the cases cited that the clinicians involved are not necessarily opposed to self-funded treatment, or treatment in a private intensive care unit, but have a particular objection to the use of limited public healthcare resources.

viii Some objections to resource-based treatment limitations are linked to specific religious beliefs (eg, some opposition to Obamacare).^16^

ix Weinstock argues that COs that jeopardise the function of a healthcare system are unreasonable, and should not be accommodated.^25^

x In the system as it stands, there would be no way for a physician to act on their conscientious belief and provide Kadcyla. If Conscientious Treatment were to be accepted, the idea is that individual physicians could override national or regional decisions not to fund a particular treatment by lodging a CO.
